# Novel Betacoronavirus in Dromedaries of the Middle East, 2013

**DOI:** 10.3201/eid2004.131769

**Published:** 2014-04

**Authors:** Patrick C.Y. Woo, Susanna K.P. Lau, Ulrich Wernery, Emily Y.M. Wong, Alan K.L. Tsang, Bobby Johnson, Cyril C.Y. Yip, Candy C.Y. Lau, Saritha Sivakumar, Jian-Piao Cai, Rachel Y.Y. Fan, Kwok-Hung Chan, Ringu Mareena, Kwok-Yung Yuen

**Affiliations:** The University of Hong Kong, Hong Kong, China (P.C.Y. Woo, S.K.P. Lau, E.Y.M. Wong, A.K.L. Tsang, C.C.Y. Yip, C.C.Y. Lau, J.-P. Cai, R.Y.Y. Fan, K.H. Chan, K.-Y. Yuen);; Central Veterinary Research Laboratory, Dubai, United Arab Emirates (U. Wernery, B. Johnson, S. Sivakumar, R. Mareena)

**Keywords:** coronavirus, dromedary, camel, Middle East, betacoronavirus, dromedaries, camel coronavirus, dromedary coronavirus, dromedary camel coronavirus UAE-HKU23, DcCoVUAE-HKU23, DcCoV, viruses, United Arab Emirates, Dubai, Middle East respiratory syndrome coronavirus, MERS-CoV, zoonoses

## Abstract

In 2013, a novel betacoronavirus was identified in fecal samples from dromedaries in Dubai, United Arab Emirates. Antibodies against the recombinant nucleocapsid protein of the virus, which we named dromedary camel coronavirus (DcCoV) UAE-HKU23, were detected in 52% of 59 dromedary serum samples tested. In an analysis of 3 complete DcCoV UAE-HKU23 genomes, we identified the virus as a betacoronavirus in lineage A1. The DcCoV UAE-HKU23 genome has G+C contents; a general preference for G/C in the third position of codons; a cleavage site for spike protein; and a membrane protein of similar length to that of other betacoronavirus A1 members, to which DcCoV UAE-HKU23 is phylogenetically closely related. Along with this coronavirus, viruses of at least 8 other families have been found to infect camels. Because camels have a close association with humans, continuous surveillance should be conducted to understand the potential for virus emergence in camels and for virus transmission to humans.

The 2003 epidemic of severe acute respiratory syndrome (SARS) boosted interest in the discovery of new coronaviruses (CoVs) (*1*�?"*3*). In 2004, a novel human CoV (HCoV), named HCoV-NL63, was reported (*4*), and the discovery of another novel HCoV, HCoV-HKU1, was described and further characterized in 2005 (5,*6*) and 2006 (*7*). SARS-CoV�?"like viruses have also been reported in Chinese horseshoe bats in Hong Kong, China, and other horseshoe bats in China (*8*,*9*). The discovery in Chinese horseshoe bats in Yunnan, China, of a new SARS-CoV�?"like virus that uses ACE2 as receptor has furthered interest in discovering animal origins of human infections (*10*). We have discovered 20 other animal CoVs that include 2 novel betacoronavirus lineages and a novel genus, *Deltacoronavirus* (*11*�?"*20*). From our studies, bats and birds were shown to be the gene sources for fueling the evolution and dissemination of alphacoronaviruses and betacoronaviruses and of gammacoronaviruses and deltacoronaviruses, respectively (*18*).

In 2012, a novel CoV, Middle East respiratory syndrome CoV (MERS-CoV) emerged as a cause of severe respiratory infections associated with high rates of death among humans; the virus is closely related to tylonycteris bat CoV HKU4 and pipistrellus bat CoV HKU5 (Pi-Bat CoV HKU5) (*21*�?"*23*). It has also been shown that dromedaries in the Middle East possess MERS-CoV neutralizing antibodies (*24*). To further knowledge of the evolution and dissemination of CoVs, we conducted a molecular epidemiology study of fecal samples obtained from dromedaries in Dubai, United Arab Emirates.

## Materials and Methods

### Samples

Dromedary fecal and serum samples used in the study were leftover specimens that had been submitted for pathogen screening (feces) or preventive health screening (serum) to Central Veterinary Research Laboratory (Dubai, United Arab Emirates) during January�?"July 2013. The fecal and serum samples were not obtained from the same animals. None of the dromedaries tested were known to have had contact with bats or horses.

We tested a total of 293 fecal samples: 232 from teenage and adult dromedaries (*Camelusdromedarius*) (>1 year of age) and 61 from dromedary calves (<1 year of age). Among the 293 samples, 6 were collected in January, 209 in February, 5 in March, 39 in April, 16 in May, 7 in June, and 11 in July 2013.

We tested a total of 59 serum samples: 55 from teenage and 4 from adult dromedaries. The serum samples were collected from female dairy farm or racing dromedaries. The dairy dromedaries were purchased from various countries (e.g., Saudi Arabia, Oman, Sudan, and Pakistan); the number of dairy dromedaries from each country and their lengths of stay in Dubai were not known. The racing dromedaries were from Dubai Emirate.

### RNA Extraction, Reverse Transcription PCR, and DNA Sequencing

Viral RNA extraction was conducted as described (*20*,*23*). Initial CoV screening was performed by amplifying a 440-bp fragment of CoV RNA-dependent RNA polymerase (RdRp) gene by using conserved primers (5�?�-GGTTGGGACTATCCTAAGTGTGA-3�?� and 5�?�-ACCATCATCNGANARDATCATNA-3�?�and degenerative bases N, R, and D, representing A/C/T/G, A/G, and A/G/T, respectively. After the novel CoV was detected in samples, subsequent screening was performed by using specific primers 5�?�-ACTATGACTGGCAGAATGTT-3�?� and 5�?�-TAATAAGGCGACGTAACATA-3�?�. To amplify a 126-bp fragment of RdRp gene, reverse transcription PCR (RT-PCR) and DNA sequencing were performed as described (*20*,*23*).

### Virus Culture

The fecal samples from 3 dromedaries tested positive for CoV. These samples were cultured in HRT-18G, Vero E6, Caco-2, and LLC-MK2 cell lines.

### Complete Genome Sequencing and Analysis

Three complete genomes of DcCoV UAE-HKU23 (265F, 362F, and 368F) were amplified and sequenced as described (*5*,*12*). RNA extracted from fecal specimens was used as template, and a database of CoV genes and genomes (CoVDB, http://covdb.microbiology.hku.hk) (*25*) was used for sequence retrieval. Sequences were assembled and edited to produce final sequences.

We used EMBOSS Needle (http://www.ebi.ac.uk/Tools/psa/emboss_needle/) to compare the nucleotide sequences of the genomes and the deduced amino acid sequences with those for other CoVs. A neighbor-joining phylogenetic tree with 1,000 bootstraps was constructed by using the Jones-Taylor-Thornton substitution model; gamma distribution among sites was conducted in MEGA5 (*26*).

### Real-time Quantitative RT-PCR

According to our protocol (*19*,*20*), all samples positive for DcCoV UAE-HKU23 by RT-PCR were subjected to quantitative RT-PCR (qRT-PCR).The assay was performed, as described (*27*), using a real-time 1-step qRT-PCR with DcCoV UAE-HKU23 primers 5�?�-ATAGCGGCTACACGTGGTGTT-3�?� and 5�?�-TCCCAGCCGCCATAAAACT-3�?� and probe 5�?�-(FAM) CTGTTGTTATAGGCACCACT (BHQ1)-3�?�. To generate calibration curves, we prepared a series of 6 log_10_ dilutions equivalent to 10^1^�?"10^6^ copies per reaction mixture and ran them in parallel with the test samples.

### Cloning and Purification

The nucleocapsid proteins of SARS-CoV (betacoronavirus lineage B), Pi-Bat CoV HKU5 (betacoronavirus lineage C), and rousettus bat coronavirus (Ro-BatCov HKU9; betacoronavirus lineage D) were obtained as described (*2*,*16*). To produce a plasmid for DcCoV UAE-HKU23 nucleocapsid protein purification, primers 5�?�-CATGCCATGGGCATGTCTTTTACTCCTGGTAAGC-3�?� and 5�?�- CCGCTCGAGTATTTCTGAGGTGTTTTCAG-3�?� were used to amplify the gene encoding the nucleocapsid protein of DcCoV UAE-HKU23. The sequence encoding aa 1�?"672 of the nucleocapsid protein was amplified and cloned into the *Nco*I and *Xho*I sites of expression vector pET-28b(+) (Merck KGaA, Darmstadt, Germany). Recombinant nucleocapsid protein was expressed and purified by using the Ni^2+^-loaded HiTrap Chelating System (GE Healthcare Life Sciences, Little Chalfont, UK) according to the manufacturer�?(tm)s instructions. The nucleocapsid protein of MERS-CoV was cloned and purified by using the method described above with primers 5�?�-GGAATTCCATATGATGGCATCCCCTGCTGCACCTC-3�?� and 5�?�- ATAAGAATGCGGCCGCATCAGTGTTAACATCAATCATT-3�?�.

### Western Blot Analysis

Western blot analysis was performed as described (*5*) with 1.5 I1/4g of purified (His)_6_-tagged recombinant nucleocapsid protein of DcCoV UAE-HKU23 and 1:2,000, 1:4,000, and 1:8,000 dilutions of dromedary serum samples. Antigen�?"antibody interaction was detected by using 1:4,000 diluted horseradish peroxidase�?"conjugated Goat Anti-Llama IgG (Life Technologies, Carlsbad, CA, USA) and the ECL fluorescence system (GE Healthcare Life Sciences).

To determine the possibility of cross-reactivity between antibodies against the nucleocapsid protein of DcCoV UAE-HKU23 and that of other betacoronavirus lineages, we tested 3 serum samples that were positive for antibody against the nucleocapsid protein of DcCoV UAE-HKU23. We used 1.5 I1/4g of purified (His)_6_-tagged recombinant nucleocapsid protein of 3 betacoronaviruses (SARS-CoV [lineage B], Pi-BatCoV HKU5 [lineage C], and Ro-BatCoV HKU9 [lineage D]) and 1:2,000 dilutions of serum samples. To determine the presence of antibodies against the nucleocapsid protein of MERS-CoV, we tested the serum samples by using 1.5 I1/4g of purified (His)_6_-tagged recombinant nucleocapsid protein of MERS-CoV and 1:2,000 dilutions of serum samples. Antigen�?"antibody interaction was detected as described above.

### Indirect Immunofluorescence

Anti�?"MERS-CoV antibody detection by indirect immunofluorescence was performed as described (*28*) with minor modifications. In brief, Vero cells infected with MERS-CoV were prepared as described (*28*). Camel serum samples were screened at a dilution of 1:160 on infected and noninfected control cells. Antigen�?"antibody interaction was detected by using fluorescein isothiocyanate�?"conjugated Goat Anti-Llama IgG (Life Technologies). Serum samples positive at a screening dilution of 1:160 were further titrated with serial 2-fold dilutions. The indirect immunofluorescence antibody titer was the highest dilution giving a positive result.

### Neutralization Antibody Test

The neutralization antibody test was performed as described (*28*). In brief, starting with a serum dilution of 1:10, we prepared serial 2-fold dilutions of serum in 96-well microtiter plates. For each serum dilution, 0.05 mL of the dilution was mixed with 0.05 mL of 200 MERS-CoV 50% tissue culture infectious doses and incubated at 37A�C for 1.5 h in a CO_2_ incubator. Then 0.1 mL of virus�?"serum mixture was inoculated in duplicate wells of 96-well microtiter plates with preformed monolayers of Vero cells and further incubated at 37A�C for 3�?"4 days. Cytopathic effects were observed by using an inverted microscope on days 3 and 4 after inoculation. The neutralizing antibody titer was determined as the highest dilution of serum that completely suppressed the cytopathic effects in at least half of the infected wells.

### Estimation of Substitution Rates and Divergence Dates

The number of synonymous substitutions per synonymous site, *Ks*, and the number of nonsynonymous substitutions per nonsynonymous site, *Ka*, for each coding region between each pair of strains were calculated by using the Nei-Gojobori method (Jukes-Cantor) in MEGA5 (*26*). Divergence time was calculated on the basis of RdRp gene sequence data by using a Bayesian Markov Chain Monte Carlo approach as implemented in BEAST version 1.8.0 (http://beast.bio.ed.ac.uk), as described (*15*,*19*,*29*,*30*). Bayesian skyline under a relaxed-clock model with an uncorrelated exponential distribution was adopted for making inferences because Bayes factor analysis for the RdRp gene indicated that this model fitted the data better than other models tested.

## Results

### Identification of CoV in Dromedaries

Of the 293 fecal samples tested, 14 (4.8%) were RT-PCR positive for the CoV RdRp gene; 1 (0.4%) of the samples was from an adult dromedary and 13 (21.3%) were from calves (Table 1). Of the 14 positive samples, 11 were collected in April and 3 were collected in May. Ten of the 14 samples were submitted to the Central Veterinary Research Laboratory for routine checking, and the other 4 were collected because the dromedaries had diarrhea. Sequencing results indicated a 126-nt sequence identical to that of equine CoV, a betacoronavirus 1 species in lineage A (betacoronavirus A1).

**Table 1:** Epidemiologic data for dromedaries in the Middle East that were positive for a novel betacoronavirus, DcCoV UAE-HKU23, 2013*

Age category	Reason for sample testing	Virus load
Calf	Routine check	3.3 A- 10^6^
Calf	Routine check	1.1 A- 10^6^
Calf	Routine check	1.5 A- 10^5^
Calf	Routine check	3.4 A- 10^5^
Calf	Routine check	3.2 A- 10^6^
Calf	Routine check	6.5 A- 10^5^
Calf	Routine check	9.5 A- 10^5^
Calf	Routine check	5.7 A- 10^4^
Calf	Routine check	2.1 A- 10^5^
Calf	Routine check	4.4 A- 10^5^
Adult	Diarrhea	7.3 A- 10^5^
Calf	Diarrhea	9.8 A- 10^7^
Calf	Diarrhea	4.4 A- 10^7^
Calf	Diarrhea	1.0 A- 10^7^

*DcCoV, dromedary camel coronavirus; calf, <1 year of age.

### Virus Culture and Virus Load

Attempts to stably passage DcCoV UAE-HKU23 in cell cultures were unsuccessful; no cytopathic effect or viral replication was detected. Real-time qRT-PCR showed that the amounts of DcCoV UAE-HKU23 RNA ranged from 5.7 A- 10^4^ copies/mL to 9.8 A- 10^7^ copies/mL (median 8.4 A- 10^5^) in the 14 fecal samples positive for DcCoV UAE-HKU23 (Table 1).

### Genome Organization and Coding Potential

**Figure 1:**
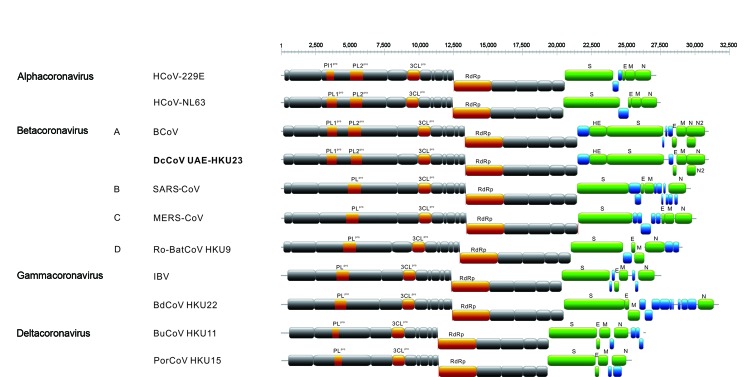
Genome organizations of a novel betacoronavirus, in boldface, discovered in dromedaries in the Middle East in 2013, and representative coronaviruses from each coronavirus genus (labeled on left). Numbers at top represent genome position. A, B, C, and D represent betacoronavirus lineages. Papain-like proteases (PL1^pro^, PL2^pro^, and PL^pro^), chymotrypsin-like protease (3CL^pro^), and RNA-dependent RNA polymerase (RdRp) are represented by orange boxes. Hemagglutinin esterase (HE) and spike (S), envelope (E), membrane (M), and nucleocapsid (N) proteins are represented by green boxes. Putative accessory proteins are represented by blue boxes. HCoV, human coronavirus; BCoV, bovine CoV; DcCoV, dromedary camel CoV; SARS-CoV, severe acute respiratory syndrome CoV; MERS-CoV, Middle East respiratory syndrome CoV; Ro-BatCoV, rousettus bat CoV; IBV, infectious bronchitis virus; BdCoV, bottlenose dolphin CoV; BuCoV, bulbul CoV; PorCoV, porcine CoV.

The 3 complete genomes of DcCoV UAE-HKU23 (GenBank accession nos. KF906249�?"KF906251) were 31,036 bases and had a G+C content of 37% (Table 2). The genome organization is similar to that of other betacoronavirus lineage A CoVs (Figure 1). Additional open-reading frames (ORFs) coding for nonstructural proteins (NSPs) NS2 and NS5 are found. DcCoV UAE-HKU23 and other CoVs in betacoronavirus lineage A possess the same putative transcription regulatory sequence motif, 5�?�-UCUAAAC-3�?�, at the 3�?� end of the leader sequence and preceding most ORFs (Table 3; Technical Appendix Table 1) (*19*,*30*).

**Table 2:** Comparison of representative coronaviruses with a novel betacoronavirus, DcCoV UAE-HKU23, discovered in dromedaries in the Middle East, 2013*

Coronavirus (genome)	Genome features			% Pairwise amino acid identity with DcCoV UAE-HKU23 (265F)				
	Size, bases	G+C content						
				3CL^pro^	RdRp	Helicase	S protein	N protein
Alphacoronavirus								
HCoV-229E	27,317	0.38		44.4	55.8	57.9	26.9	25.1
HCoV-NL63	27,553	0.34		42.8	56.0	57.5	26.4	24.1
Betacoronavirus								
Lineage A								
BCoV	31,028	0.37		99.7	99.9	99.5	94.1	98.4
SACoV	30,995	0.37		99.7	99.8	99.3	94.1	98.9
DcCoV UAE-HKU23 (362F)	31,036	0.37		99.7	100	100	99.8	100
DcCoV UAE-HKU23 (368F)	31,036	0.37		99.7	100	100	99.8	100
Lineage B								
SARS-CoV	29,751	0.41		48.4	66.3	68.0	30.4	34.4
Lineage C								
MERS-CoV	30,119	0.41		53.3	68.2	67.9	31.8	34.7
Lineage D								
Ro-BatCoV HKU9	29,114	0.41		48.4	66.5	67.5	29.2	32.5
Gammacoronavirus								
Infectious bronchitis virus	27,608	0.38		42.1	60.6	60.2	24.8	28.0
BdCoV HKU22	31,750	0.39		45.3	60.0	58.2	25.7	28.9
Deltacoronavirus								
BuCoV HKU11	26,476	0.39		38.5	51.2	47.5	26.4	23.8
PorCoV HKU15	25,421	0.43		38.8	51.8	48.6	26.0	23.1

*The representative coronaviruses were those with complete genomes sequences available. DcCoV, dromedary camel coronavirus; 3CL^pro^, chymotrypsin-like protease; RdRp, RNA-dependent RNA polymerase; S protein, spike protein; N protein, nucleocapsid protein; HCoV, human CoV; BCoV, bovine CoV; SACoV, sable antelope CoV; SARS-CoV, severe acute respiratory syndrome CoV; MERS-CoV, Middle East respiratory syndrome CoV; Ro-BatCoV, rousettus bat CoV; BdCoV, bottlenose dolphin CoV; BuCoV, bulbul CoV; PorCoV, porcine CoV.

**Table 3:** Putative transcription regulatory sequence of betacoronavirus A1 members*

ORF	Putative transcription regulatory sequence (distance, in bases, to AUG)�?							
	DcCoV UAE-HKU23	BCoV	CRCoV	SACoV	GiCoV	PHEV	HCoV-OC43	ECoV
1ab	UCUAAAC (140)	UCUAAAC (140)	UCUAAAC (140)	UCUAAAC (140)	UCUAAAC (140)	UCUAAAC (140)	UCUAAAC (140)	UCUAAAC (140)
NS2	UCUAAAC (7)	UCUAAAC (7)	UCUAAAC (7)	UCUAAAC (7)	UCUAAAC (7)	UCUAAAC (1)	UCUAAAC (7)	UCUAAAA (12)
HE	ACUAAAC (9)	ACUAAAC (9)	ACUAAAC (9)	ACUAAAC (9)	ACUAAAC (9)	ACUAAAC (9)	AUUAAAC (9)	UCUAAAC (9)
S	UCUAAAC (0)	UCUAAAC (0)	UCUAAAC (0)	UCUAAAC (0)	UCUAAAC (0)	UCUAAAC (0)	UCUAAAC (0)	UCUAAAC (0)
NS5	GGUGAAC (51)	GGU**AG**AC (**50**)	GGU**AG**AC (51)	GGU**AG**AC (**50**)	GGU**AG**AC (**50**)	**UUAAGCA** (**32**)	**UCUAGCA** (**20/32**)	**UAUACUUUAUAA** (**41**)
E	UCCAAAC (123)	UCCAAAC (123)	UCCAAAC (123)	UCCAAAC (123)	UCCAAAC (123)	UCCAAAC (123)	UCCAAAC (123)	UCCAAAC (123)
M	UCCAAAC (3)	UCCAAAC (3)	UCCAAAC (3)	UCCAAAC (3)	UCCAAAC (3)	UCCAAAC (3)	UCCAAAC (3)	UCCAAAC (3)
N	UCUAAAC (7)	UCUAAAC (7)	UCUAAAC (7)	UCUAAAC (7)	UCUAAAC (7)	UCUAAAC (7)	UCUAAAU (7)	UCUAAAC (7)

*ORF, open reading frame; DcCoV, dromedary camel coronavirus; BCoV, bovine CoV; CRCoV, canine respiratory CoV; SACoV, sable antelopeCoV; GiCoV, giraffe CoV; PHEV, porcine hemagglutinating encephalomyelitis virus; HCoV, human CoV; ECoV, equine CoV; NS, nonstructural protein; HE, hemagglutinin esterase; S, spike protein; E, envelope protein; M, membrane protein; N, nucleocapsid protein.�?"�? Boldface indicates bases and distances to AUG that are different from those for DcCoV UAE-HKU23.

The characteristics of putative NSPs in ORF1ab of DcCoV UAE-HKU23 are shown in Technical Appendix Table 2. The ORF1ab polyprotein shared 70.7%�?"99.3% aa identity with polyproteins of other betacoronavirus lineage A CoVs. The predicted putative cleavage sites were conserved between DcCoV UAE-HKU23 and other members of betacoronavirus A1 of the *Betacoronavirus* genus. The lengths of NSPs 1�?"3, 13, and 15 in DcCoV UAE-HKU23 differed from those in equine CoV, porcine hemagglutinating encephalomyelitis virus, and/or HCoV-OC43 as a result of deletions/insertions.

**Figure 2:**
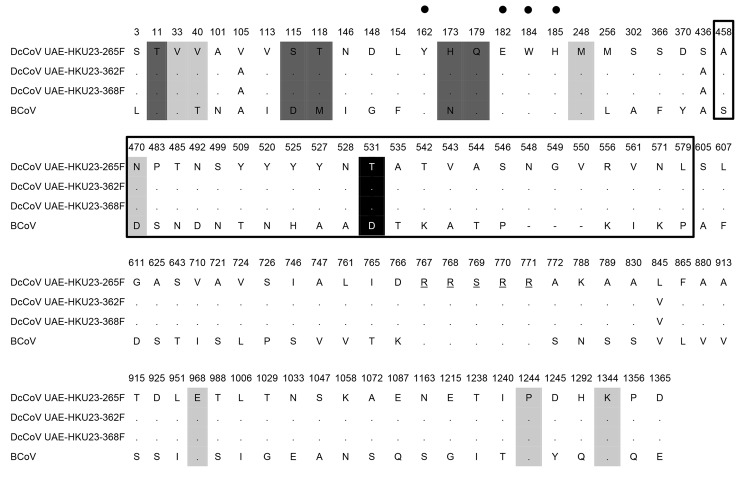
Amino acid comparison of the spike protein of a novel betacoronavirus, dromedary camel coronavirus (DcCoV) UAE-HKU23, discovered in dromedaries in the Middle East in 2013, with that of bovine coronavirus (BCoV; GenBank accession no. AF391541).Amino acid substitution sites, key amino acids for virulence and receptor binding in BCoV, and cleavage sites are shown. Amino acid positions are given with reference to DcCoV UAE-HKU23. Conserved amino acids, compared with those of DcCoV UAE-HKU23 (strain 265F), are represented by dots. Amino acids of putative cleavage sites are underlined. Amino acids within the S1 hypervariable region of BCoV are marked with open boxes. Amino acid sites central to virulence in BCoV are highlighted in light gray. Amino acid sites shown to affect S1-mediated receptor binding in BCoV are highlighted in dark gray. The 4 critical sugar-binding residues are indicated by black dots. The amino acid site that discriminated between enteric and respiratory BCoV strains is highlighted in black.

The amino acid sequence of the predicted spike protein of DcCoV UAE-HKU23 is most similar to that of bovine coronavirus (BCoV) and sable antelope CoV, with which DcCoV UAE-HKU23 has 94.1% similarity (Table 2). A comparison of the amino acid sequences of DcCoV UAE-HKU23 spike protein and BCoV spike protein showed 81 aa polymorphisms, of which 24 were seen within the region previously identified as hypervariable among the spike protein of other betacoronavirus lineage A CoVs (*31*) (Figure 2); this finding suggests that this region in DcCoV UAE-HKU23 is also subject to strong immune selection. BCoV has been found to use *N*-acetyl-9-*O*acetyl neuramic acid as a receptor for initiation of infection (*32*). Among the 5 aa that may affect S1-mediated receptor binding in BCoV (*31*), 2 aa (threonine at position 11 and glutamine at position 179) were conserved in DcCoV UAE-HKU23 (Figure 2). However, at positions 115, 118, and 173, aspartic acid, methionine, and asparagine observed in BCoV and were replaced by serine, threonine, and histidine, respectively, in DcCoV UAE-HKU23. A recent report identified 4 aa acids that were critical sugar-binding residues in the spike protein of BCoV (tyrosine, glutamic acid, tryptophan, and histidine at positions 162, 182, 184, and 185, respectively) (*32*); all 4 aa were also present in spike protein of DcCoV UAE-HKU23 (Figure 2). Another study identified 7 aa substitutions in the spike protein of BCoV that differed between virulent and avirulent, cell culture-adapted strains (*31*); 5 of the 7 aa from virulent strains were also conserved in DcCoV UAE-HKU23, and amino acid substitutions were observed in the other 2 aa (threonine�+'valine at position 40 and aspartic acid�+'asparagine at position 470). It has also been reported that an amino acid change at position 531 of the spike protein of BCoV discriminated between enteric (aspartic acid/asparagine) and respiratory (glycine) strains (*33*). At this position, a threonine was conserved in all 3 genomes of DcCoV UAE-HKU23.

NS5 of DcCoV UAE-HKU23 shares 83.5%�?"98.2% aa identity with the corresponding NSPs of betacoronavirus A1 members. In murine hepatitis virus, translation of the envelope protein is cap-independent, through an internal ribosomal entry site (*19*,*30*). However, a preceding transcription regulatory sequence, 5�?�-UCCAAAC-3�?�, can be identified upstream of the envelope protein in DcCoV UAE-HKU23, as in other betacoronavirus A1 members (Table 3, Technical Appendix Table 1) (*19*,*30*). Downstream to nucleocapsid protein, the 3�?�-untranslated region contains a predicted bulged stem-loop structure of 68 nt (nt position 30,747�?"30,814) that is conserved in betacoronaviruses (*34*). Overlapping with the bulged stem-loop structure by 5 nt, is a conserved pseudoknot structure (nt position 30810�?"30863) that is important for CoV replication.

### Phylogenetic Analyses

**Figure 3:**
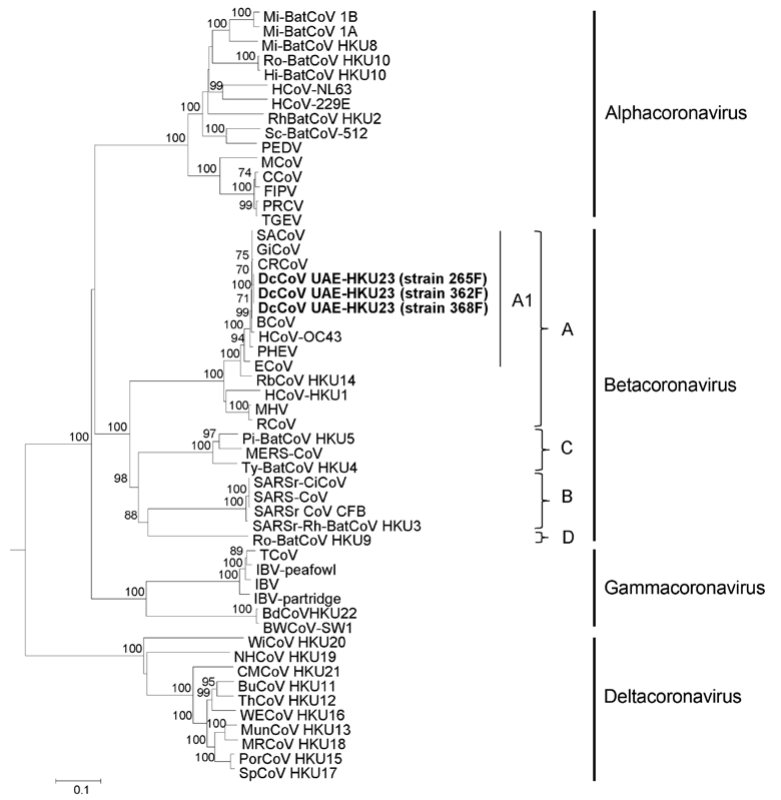
Phylogenetic analysis of open reading frame (ORF) 1b polyprotein of dromedary camel coronavirus (DcCoV) UAE-HKU23 from dromedaries of the Middle East, 2013. The tree was constructed by the neighbor-joining method, using Jones-Taylor-Thornton substitution model with gamma distributed rate variation and bootstrap values calculated from 1,000 trees. Bootstrap values of <70% are not shown. A total of 2,725 aa positions in ORF1b polyprotein were included in the analysis. The tree was rooted to Breda virus (GenBank accession no. AY_427798). Betacoronavirus lineages A1 and A�?"D are indicated on the right. Boldface indicates the 3 strains of DcCoV UAE-HKU23 characterized in this study. Virus definitions and GenBank accession numbers (in parentheses) follow: Mi-BatCoV 1B, miniopterus bat CoV 1B (NC_010436); Mi-BatCoV 1A (NC_010437); Mi-BatCoV HKU8 (NC_010438); Ro-BatCoV HKU10, rousettus bat CoV HKU10 (JQ989270); Hi-BatCoV HKU10, hipposideros bat CoV HKU10 (JQ989266); HCoV-NL63, human CoV NL63 (NC_005831); HCoV-229E (NC_002645); RhBatCoV HKU2, rhinolophus bat CoV HKU2 (EF203064); Sc-BatCoV-512, scotophilus bat CoV 512 (NC_009657); PEDV, porcine epidemic diarrhea virus (NC_003436);MCoV, mink CoV (HM245925);CCoV, canine CoV (GQ477367); FIPV, feline infectious peritonitis virus (AY994055); PRCV, porcine respiratory CoV; TGEV, transmissible gastroenteritis virus; SACoV, sable antelope CoV; GiCoV, giraffe CoV (EF424622); CRCoV, canine respiratory CoV (JX860640); BCoV, bovine CoV (NC_003045); HCoV-OC43, human CoV OC43 (NC_005147);PHEV, porcine hemagglutinating encephalomyelitis virus (NC_007732); ECoV, equine CoV (NC_010327); RbCoV HKU14, rabbit CoV HKU14 (JN874559); HCoV-HKU1, human CoV HKU1 (NC_006577); MHV, murine hepatitis virus (NC_001846); RCoV, rat CoV (NC_012936); Pi-BatCoV HKU5, pipistrellus bat CoV HKU5 (NC_009020); MERS-CoV, Middle East respiratory syndrome CoV (JX869059);Ty-BatCoV HKU4, tylonycteris bat CoV HKU4 (NC_009019); SARSr-CiCoV, SARS-related palm civet CoV (AY304488); SARS-CoV, severe acute respiratory syndrome�?"associated human CoV (NC_004718); SARSrCoV CFB, SARS-related Chinese ferret badger CoV (AY545919); SARS-related rhinolophus bat CoV HKU3 (DQ022305); Ro-BatCoV HKU9, rousettus bat CoV HKU9 (NC_009021); TCoV, turkey CoV (NC_010800); IBV-peafowl, peafowl CoV (AY641576); IBV, infectious bronchitis virus (NC_001451); IBV-partridge, partridge CoV (AY646283); BdCoV HKU22, bottlenose dolphin CoV HKU22 (KF793824); BWCoV-SW1, Beluga whale CoV SW1 (NC_010646); WiCoV HKU20, wigeon CoV HKU20 (JQ065048); NHCoV HKU19, night-heron CoV HKU19 (JQ065047); CMCoV HKU21, common-moorhen CoV HKU21 (JQ065049); BuCoV HKU11, bulbul CoV HKU11 (FJ376619); ThCoV HKU12, thrush CoV HKU12 (FJ376621); WECoV HKU16, white-eye CoV HKU16 (JQ065044); MunCoV HKU13, munia CoV HKU13 (FJ376622); MRCoV HKU18, magpie�?"robin CoV HKU18 (JQ065046); PorCoV HKU15, porcine CoV HKU15 (JQ065042); SpCoV HKU17, sparrow CoV HKU17 (JQ065045). Numbers at nodes represent bootstrap values. Scale bar indicates the estimated number of substitutions per 10 aa.

Phylogenetic trees constructed by using the amino acid sequences of ORF1b polyprotein, spike protein, and nucleocapsid protein of DcCoV UAE-HKU23 and other CoVs are shown in Figures 3�?"5. The pairwise amino acid identities of chymotrypsin-like protease (3CL^pro^), RdRp, helicase, spike protein, and nucleocapsid protein are shown in Table 2. In all 3 phylogenetic trees, DcCoV UAE-HKU23 clustered with other members of betacoronavirus A1, including BCoV, sable antelope CoV, equine CoV, HCoV-OC43, giraffe CoV, porcine hemagglutinating encephalomyelitis virus, canine respiratory CoV, and rabbit CoV (RbCoV) HKU14 (Figure 3�?"5).

**Figure 4:**
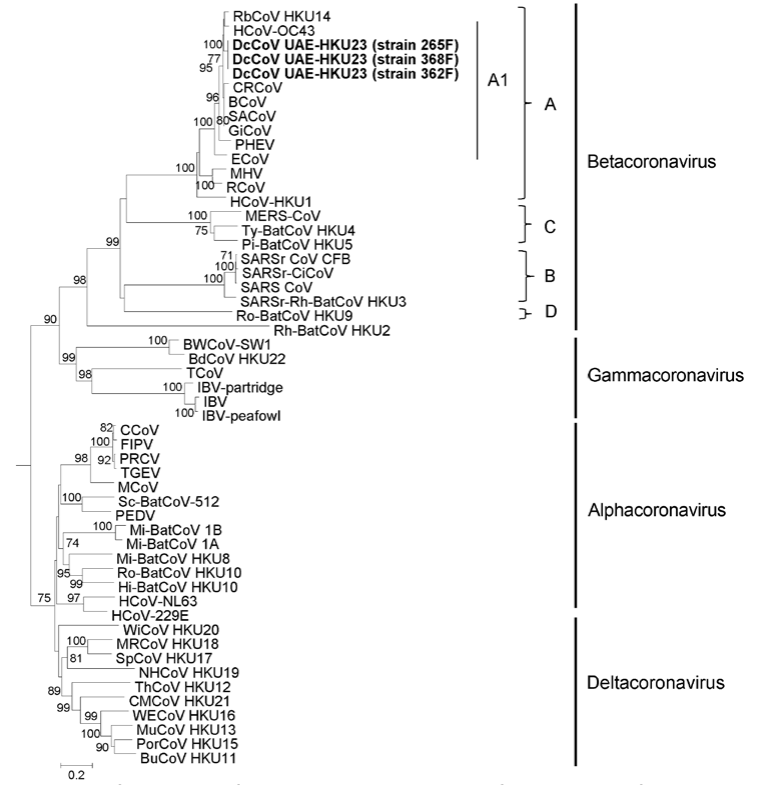
Phylogenetic analyses of spike protein of dromedary camel coronavirus (DcCoV) UAE-HKU23from dromedaries of the Middle East, 2013.The tree was constructed by the neighbor-joining method, using Jones-Taylor-Thornton substitution model with gamma distributed rate variation and bootstrap values calculated from 1,000 trees. Bootstrap values of <70% are not shown. A total of 1,366 aa positions in spike protein were included in the analysis. The tree was rooted to Breda virus (GenBank accession no. AY_427798).Betacoronavirus lineages A1 and A�?"D are indicated on the right. Boldface indicates the 3 strains of DcCoV UAE-HKU23 characterized in this study. Virus definitions and GenBank accession numbers (in parentheses) follow: RbCoV HKU14, rabbit CoV HKU14 (JN874559); HCoV-OC43, human CoV OC43 (NC_005147); CRCoV, canine respiratory CoV (JX860640); BCoV, bovine CoV (NC_003045); SACoV, sable antelope CoV (EF424621); GiCoV, giraffe CoV (EF424622); PHEV, porcine hemagglutinating encephalomyelitis virus (NC_007732); ECoV, equine CoV (NC_010327); MHV, murine hepatitis virus (NC_001846); RCoV, rat CoV (NC_012936); HCoV-HKU1, human CoV HKU1 (NC_006577); MERS-CoV, Middle East respiratory syndrome CoV (JX869059); Ty-BatCoV HKU4, tylonycteris bat CoV HKU4 (NC_009019); Pi-BatCoV HKU5, pipistrellus bat CoV HKU5 (NC_009020); SARSrCoV CFB, SARS-related Chinese ferret badger CoV (AY545919); SARSr-CiCoV, SARS-related palm civet CoV (AY304488); SARS-CoV, severe acute respiratory syndrome�?"associated human CoV (NC_004718); SARSr-Rh-BatCoV HKU3, SARS-related rhinolophus bat CoV HKU3 (DQ022305); Ro-BatCoV HKU9, rousettus bat CoV HKU9 (NC_009021); RhBatCoV HKU2, rhinolophus bat CoV HKU2 (EF203064); BWCoV-SW1, Beluga whale CoV SW1 (NC_010646); BdCoV HKU22, bottlenose dolphin CoV HKU22 (KF793824); TCoV, turkey CoV (NC_010800); IBV-partridge, partridge CoV (AY646283); IBV, infectious bronchitis virus (NC_001451); IBV-peafowl, peafowl CoV (AY641576); CCoV, canine CoV (GQ477367); FIPV, feline infectious peritonitis virus (AY994055); PRCV, porcine respiratory CoV (DQ811787); TGEV, transmissible gastroenteritis virus (DQ811789); MCoV, mink CoV (HM245925); Sc-BatCoV-512, scotophilus bat CoV 512 (NC_009657); PEDV, porcine epidemic diarrhea virus (NC_003436); Mi-BatCoV 1B, miniopterus bat CoV 1B (NC_010436); Mi-BatCoV 1A, miniopterus bat CoV 1A (NC_010437); Mi-BatCoV HKU8, miniopterus bat CoV HKU8 (NC_010438); Ro-BatCoV HKU10, rousettus bat CoV HKU10 (JQ989270); Hi-BatCoV HKU10, hipposideros bat CoV HKU10 (JQ989266); HCoV-NL63, human CoV NL63 (NC_005831); HCoV-229E, human CoV 229E (NC_002645); WiCoV HKU20, wigeon CoV HKU20 (JQ065048); MRCoV HKU18, magpie�?"robin CoV HKU18 (JQ065046); SpCoV HKU17, sparrow CoV HKU17 (JQ065045); NHCoV HKU19, night-heron CoV HKU19 (JQ065047); ThCoV HKU12, thrush CoV HKU12 (FJ376621); CMCoV HKU21, common-moorhen CoV HKU21 (JQ065049); WECoV HKU16, white-eye CoV HKU16 (JQ065044); MunCoV HKU13, munia CoV HKU13 (FJ376622); PorCoV HKU15, porcine CoV HKU15 (JQ065042); BuCoV HKU11, bulbul CoV HKU11 (FJ376619). Numbers at nodes represent bootstrap values. Scale bar indicates the estimated number of substitutions per 5 aa.

**Figure 5:**
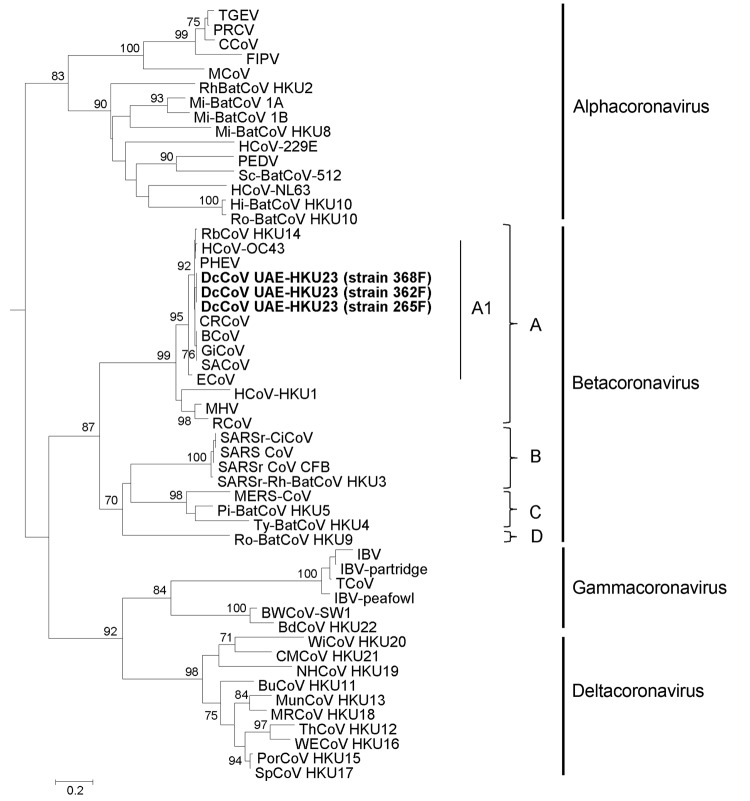
Phylogenetic analyses of the nucleocapsid protein of a novel coronavirus (CoV), dromedary camel CoV (DcCoV) UAE-HKU23, discovered in dromedaries of the Middle East, 2013.The tree was constructed by the neighbor-joining method, using Jones-Taylor-Thornton substitution model with gamma distributed rate variation and bootstrap values calculated from 1,000 trees. Bootstrap values of <70% are not shown. A total of 448 aa positions were included in the analysis. The tree was rooted to Breda virus (GenBank accession no. AY_427798). Betacoronavirus lineages A1 and A�?"D are indicated on the right. Boldface indicates the 3 strains of DcCoV UAE-HKU23 characterized in this study. Virus definitions and GenBank accession numbers (in parentheses) follow: TGEV, transmissible gastroenteritis virus (DQ811789); PRCV, porcine respiratory CoV (DQ811787); CCoV, canine CoV (GQ477367); FIPV, feline infectious peritonitis virus (AY994055); MCoV, mink CoV (HM245925); RhBatCoV HKU2, rhinolophus bat CoV HKU2 (EF203064); Mi-BatCoV 1A, miniopterus bat CoV 1A (NC_010437); Mi-BatCoV 1B, miniopterus bat CoV 1B (NC_010436); Mi-BatCoV HKU8, miniopterus bat CoV HKU8 (NC_010438); HCoV-229E, human CoV 229E (NC_002645); PEDV, porcine epidemic diarrhea virus (NC_003436); Sc-BatCoV-512, scotophilus bat CoV 512 (NC_009657); HCoV-NL63, human CoV NL63 (NC_005831); Hi-BatCoV HKU10, hipposideros bat CoV HKU10 (JQ989266); Ro-BatCoV HKU10, rousettus bat CoV HKU10 (JQ989270); RbCoV HKU14, rabbit CoV HKU14 (JN874559); HCoV-OC43, human CoV OC43 (NC_005147); PHEV, porcine hemagglutinating encephalomyelitis virus (NC_007732); CRCoV, canine respiratory CoV (JX860640); BCoV, bovine CoV (NC_003045); GiCoV, giraffe CoV (EF424622); SACoV, sable antelope CoV (EF424621); ECoV, equine CoV (NC_010327); HCoV-HKU1, human CoV HKU1 (NC_006577); MHV, murine hepatitis virus (NC_001846); RCoV, rat CoV (NC_012936); SARSr-CiCoV, SARS-related palm civet CoV (AY304488); SARS-CoV, severe acute respiratory syndrome�?"associated human CoV (NC_004718); SARSrCoV CFB, SARS-related Chinese ferret badger CoV (AY545919); SARSr-Rh-BatCoV HKU3, SARS-related rhinolophus bat CoV HKU3 (DQ022305); MERS-CoV, Middle East respiratory syndrome CoV (JX869059); Pi-BatCoV HKU5, pipistrellus bat CoV HKU5 (NC_009020); Ty-BatCoV HKU4, tylonycteris bat CoV HKU4 (NC_009019); Ro-BatCoV HKU9, rousettus bat CoV HKU9 (NC_009021); IBV, infectious bronchitis virus (NC_001451); IBV-partridge, partridge CoV (AY646283); TCoV, turkey CoV (NC_010800); IBV-peafowl, peafowl CoV (AY641576); BWCoV-SW1, Beluga whale CoV SW1 (NC_010646); BdCoV HKU22, bottlenose dolphin CoV HKU22 (KF793824); WiCoV HKU20, wigeon CoV HKU20 (JQ065048); CMCoV HKU21, common-moorhen CoV HKU21 (JQ065049); NHCoV HKU19, night-heron CoV HKU19 (JQ065047); BuCoV HKU11, bulbul CoV HKU11 (FJ376619); MunCoV HKU13, munia CoV HKU13 (FJ376622); MRCoV HKU18, magpie�?"robin CoV HKU18 (JQ065046); ThCoV HKU12, thrush CoV HKU12 (FJ376621); WECoV HKU16, white-eye CoV HKU16 (JQ065044); PorCoV HKU15, porcine CoV HKU15 (JQ065042); SpCoV HKU17, sparrow CoV HKU17 (JQ065045). Numbers at nodes represent bootstrap values. Scale bar indicates the estimated number of substitutions per 5 aa.

### Antibody Detection

**Figure 6:**
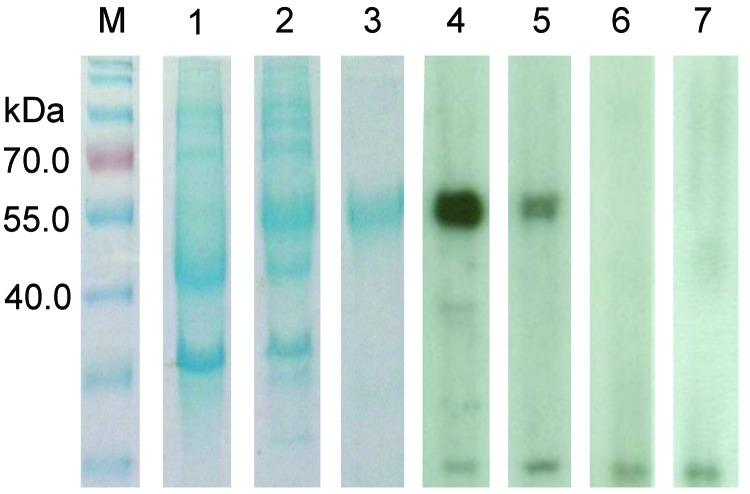
Sodium dodecyl sulfate-polyacrylamide gel electrophoresis and Western blot analysis of a novel coronavirus, dromedary camel coronavirus UAE-HKU23, discovered in dromedaries of the Middle East, 2013.Nucleocapsid protein was expressed in *Escherichia coli*. M, protein molecular-mass marker; kDa, kilodaltons. Lanes: 1, non-induced crude *E. coli* cell lysate; 2, induced crude *E. coli* cell lysate of DcCoV UAE-HKU23 nucleocapsid protein; 3, purified recombinant DcCoV UAE-HKU23 nucleocapsid protein; 4, dromedary camel serum sample strongly positive for antibody against nucleocapsid protein; 5, dromedary camel serum sample moderately positive for antibody against nucleocapsid protein; 6 and 7: dromedary camel serum sample negative for antibody against nucleocapsid protein.

Nucleocapsid protein of DcCoV UAE-HKU23 was purified. Prominent immunoreactive bands were visible for 31 (52%) of 59 dromedary serum samples; 25 of the 31 samples had titers of 2,000, three had titers of 4,000, and 3 had titers of 8,000 (Figure 6). Serum samples for all 4 adult dromedaries were positive for DcCoV UAE-HKU23 antibodies, and 27 (49%) of the 55 samples for teenage dromedaries were positive. Band sizes were �%^50 kDa, consistent with the expected size of 50.4 kDa for the full-length (His)_6_-tagged recombinant nucleocapsid protein. Only very faint bands were observed when the 3 serum samples positive for DcCoV UAE-HKU23 antibodies were incubated with nucleocapsid proteins of SARS-CoV, Pi-BatCoV HKU5, or Ro-BatCoV HKU9, indicating minimal cross-reactivity. This finding concurs with our previous observation that minimal cross-reactivity occurs between CoVs in different lineages in betacoronavirus (*16*). For MERS-CoV antibody testing, results were positive for 57 (97%) of the 59 samples by Western blot analysis, for all 59 samples by indirect immunofluorescence, and for 58 (98%) of the 59 samples by neutralization antibody test (Table 4).

**Table 4:** Detection of antibodies to MERS-CoV in dromedaries in the Middle East, 2013*

Test, antibody titer	No. (%) positive samples
Indirect immunofluorescence	
160	2 (3.4)
320	12 (20.3)
640	16 (27.1)
1280	11 (18.6)
2560	14 (23.7)
5120	4 (6.8)
Neutralization antibody test	
<10	1 (1.7)
10	7 (11.9)
20	14 (23.7)
40	28 (47.5)
80	8 (13.6)
160	1 (1.7)

*MERS-CoV, Middle East respiratory syndrome coronavirus.

### Estimation of Substitution Rates and Divergence Dates

The *Ka*, *Ks*, and *Ka*/*Ks* of the various coding regions in DcCoV UAE-HKU23 are shown in Table 5. The *Ka*/*Ks* of all the coding regions in DcCoV UAE-HKU23 was <0.5.

**Table 5:** Estimates of nonsynonymous and synonymous substitution rates in the genomes of a novel betacoronavirus, DcCoV UAE-HKU23, discovered in dromedaries of the Middle East, 2013*

Gene	*Ka*	*Ks*	*Ka*/*Ks*
NSP1	0	0.004	0
NSP2	0	0.002	0
NSP3	0.001	0.006	0.167
NSP4	0.001	0.007	0.143
NSP5	0.001	0.003	0.333
NSP6	0.001	0.003	0.333
NSP7	0	0	�?"
NSP8	0	0.016	0
NSP9	0	0	�?"
NSP10	0	0	�?"
NSP11	0	0	�?"
NSP12	0	0.002	0
NSP13	0	0.002	0
NSP14	0.001	0	�?"
NSP15	0.001	0	�?"
NSP16	0	0	�?"
NS2	0.001	0.008	0.125
HE	0.001	0.002	0.5
Spike	0.001	0.004	0.25
NS5	0	0.009	0
Envelope	0	0	�?"
Membrane	0	0	�?"
Nucleocapsid	0	0	�?"
N2	0	0	�?"

DcCoV, dromedary camel coronavirus; *Ka*, nonsynonymous site; *Ks*, synonymous site; NSP, nonstructural protein; NS, nonstructural; HE, hemagglutinin esterase; N, nucleocapsid.

By using the uncorrelated relaxed clock model on RdRp gene sequences, we estimated the date of divergence between DcCoV UAE-HKU23 and BCoV to be �%^46 years ago. We estimated that the 3 strains of DcCoV UAE-HKU23 diverged from their most recent common ancestor in March 2010 (the 95% highest posterior density interval, August 2006�?" September 2012) (Technical Appendix Figure 1).

## Discussion

We discovered a novel CoV, but no MERS-CoV, in dromedaries from the Middle East. Dromedaries are 1 of 2 surviving camel species. Dromedaries (*C. dromedarius*; 1-humped camels) inhabit the Middle East and northern and northeastern Africa; Bactrian camels (*C. bactrianus*, 2-humped camels) inhabit Central Asia. Among the 20 million camels on earth, 90% are dromedaries. In 2012, there were �%^360,000 dromedaries in the United Arab Emirates.

In this study, we discovered a novel CoV, DcCoV UAE-HKU23, from 4.8% of 293 fecal samples collected from dromedaries in Dubai. The positive samples were not collected from the same farm or stable. Moreover, there was >0.2% nt difference among the 3 complete genomes sequenced, indicating that the positive samples were not collected from a clonal outbreak. In our study, 21.3% of dromedary calves, but only 0.4% of adult dromedaries, were RT-PCR positive for DcCoV UAE-HKU23; this finding indicates that dromedary calves are probably more susceptible than adult dromedaries to infection with DcCoV UAE-HKU23. Furthermore, DcCoV UAE-HKU23 is probably stably evolving in dromedaries because the *Ka*/*Ks* of all the coding regions in the genome were <0.5. In this study, 4 of the 12 positive samples were collected from dromedaries with diarrhea. A previous report also described the presence of a betacoronavirus in the fecal sample of a dromedary calf with diarrhea (*35*). This finding raises the question of the pathologic significance of DcCoV UAE-HKU23 for camelids and warrants further animal studies.

Our serologic data showed little cross-reactivity between DcCoV UAE-HKU23 and SARS-CoV, Pi-BatCoV HKU5, and Ro-BatCoV HKU9. This finding is in line with findings from our previous studies of Ro-BatCoV HKU9, which also showed minimal serologic cross-reactivity among the 4 lineages of betacoronaviruses (*16*). These results suggest that there should be minimal cross-reactivity between DcCoV UAE-HKU23 and MERS-CoV, which belong to 2 different CoV lineages. Because we showed an extremely high prevalence of MERS-CoV antibodies in the serum samples by Western blot analysis, indirect immunofluorescence, and neutralization antibody testing, concurring with findings in a previous study (*24*), we would also expect a similar high prevalence of DcCoV UAE-HKU23 antibodies if there was major serologic cross-reactivity between MERS-CoV and DcCoV UAE-HKU23. However, our serologic data only revealed the presence of DcCoV UAE-HKU23 antibodies in 52% of the serum samples, indicating that no correlation exists between seropositivity to DcCoV UAE-HKU23 and seropositivity to MERS-CoV. Furthermore, we found no correlation between seropositivity to DcCoV UAE-HKU23 and MERS-CoV antibody titers.

In this study, correlation between DcCoV UAE-HKU23 RT-PCR positivity and seropositivity also cannot be ascertained because the fecal samples and serum samples were collected from different dromedaries. Because MERS-CoV was not present in dromedaries in the present study, an intensive search in dromedaries and other animals in other locations in the Middle East would be helpful in the search for the animal source of MERS-CoV.

**Figure 7:**
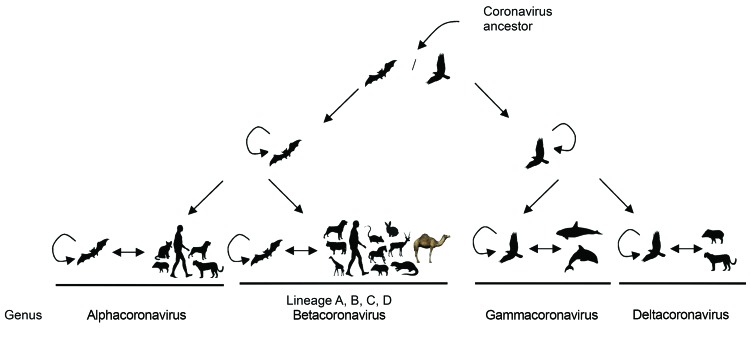
The evolution of corona viruses from their ancestors in bat and bird hosts to new virus species that infect other animals.

DcCoV UAE-HKU23 is a member of betacoronavirus A1 (Figure 7). Comparison of the amino acid identities of the 7 conserved replicase domains for species demarcation (i.e., ADP-ribose 1�?3-phosphatase, NSP5 [3CL^pro^], NSP12 [RdRp], NSP13 [helicase], NSP14 [ExoN], NSP15 [NendoU], and NSP16 [O-MT]) (*36*) between DcCoV UAE-HKU23 and other CoVs of betacoronavirus A1 revealed that in all 7 domains, the amino acid sequences of DcCoV UAE-HKU23 and other betacoronavirus A1 members shared >90% identity. This finding indicates that DcCoV UAE-HKU23 should be a member of betacoronavirus A1.

Furthermore, the genome characteristics of DcCoV UAE-HKU23 showed features similar to those of other betacoronavirus A1 members. The genomes of all betacoronavirus A1 members have G+C contents of 0.37 and the genome of RbCoV HKU14, a recently discovered CoV closely related to betacoronavirus A1 (*19*), has a G+C content of 0.38. This G+C content differs substantially from those of other CoV species of lineage A betacoronaviruses, which have G+C contents of 0.32 (HCoV-HKU1), 0.41 (rat CoV) and 0.42 (murine hepatitis virus) (Table 2). The difference in the genome characteristics between A1 and non-A1 members of betacoronavirus is also reflected by their codon usage bias, in which the general preference of using G/C in the third position of the codons decreases from murine hepatitis virus and rat CoV to betacoronavirus A1 members and RbCoV HKU14 to HCoV-HKU1 (Technical Appendix Figure 2). The cleavage site for spike protein of betacoronavirus A1 members is RRS/QRR, whereas those of HCoV-HKU1, RbCoV HKU14, and murine hepatitis virus are RRKRR, LRSRR, and RAR/H/DR/S, respectively. The length of membrane genes for betacoronavirus A1 members and RbCoV HKU14 is 693 bases, whereas the lengths for HCoV-HKU1 and murine hepatitis virus are 672 and 687 bases, respectively.

DcCoV UAE-HKU23 is phylogenetically closely related to other betacoronavirus A1 members, which in turn are closely related to other CoVs of betacoronavirus lineage A. Despite their close relationships, no recombination was detected between DcCoV UAE-HKU23 and other betacoronavirus A1 members by bootscan analysis (data not shown). These CoVs of betacoronavirus A1 may be using different receptors in their corresponding hosts because their spike protein is one of the proteins that show the largest difference among different CoVs. Most of the differences among the spike proteins in different betacoronavirus A1 members were also observed in the N terminal half of their spike protein, where the receptor binding domains should be located.

Camels are one of the most unique mammals on earth. In particular, they have shown perfect adaptation to desert life, which presents temperature extremes and a scarce supply of food and water. In the past, camels were used for transportation of humans and goods and for military uses. Moreover, for humans, they provide a good source of meat, milk, and wool. Camels are also important recreational animals in the Middle East and are used for camel racing. Having been associated with humans for at least 5,000 years, camels usually pose little physical danger to humans. However, infectious pathogens, such as brucellosis, can occasionally be transmitted from camels to humans. Apart from the present novel CoV, viruses of at least 8 taxonomic families (i.e., *Paramyxoviridae*, *Flaviviridae*, *Herpesviridae*, *Papillomaviridae*, *Picornaviridae*, *Poxviridae*, *Reoviridae*, and *Rhabdoviridae*) have been found to infect camels (*37*�?"*39*). Because camels are closely associated with humans, continuous surveillance of viruses in this hardy group of animals is needed to understand the potential for virus emergence and transmission to humans.

Technical Appendix Coding potential and putative transcription regulatory sequences of the genomes of dromedary camel coronavirus (DcCoV) UAE-HKU23; characteristics of putative nonstructural proteins of open-reading frame (ORF) 1ab in DcCoV UAE-HKU23; estimation of tMRCA of DcCoV UAE-HKU23 strains, DcCoV UAE-HKU23 strains/BCoV and DcCoV UAE-HKU23 strains/BCoV/HCoV-OC43; and a plot of effective number of codons against use of G or C at third position of codons of ORF1ab, HE, S, E, M and N genes in betacoronavirus lineage A.
