# Supplementary material for: Novel Betacoronavirus in Dromedaries of the Middle East, 2013
Source: Emerg Infect Dis. 2014 Apr;20(4):560–72. doi: 10.3201/eid2004.131769 (PMC3966378; doi:10.3201/eid2004.131769)
Supplement: Technical Appendix — Coding potential and putative transcription regulatory sequences of the genomes of dromedary camel coronavirus (DcCoV) UAE-HKU23; characteristics of putative nonstructural proteins of open-reading frame (ORF) 1ab in DcCoV UAE-HKU23; estimation of tMRCA of DcCoV UAE-HKU23 strains, DcCoV UAE-HKU23 strains/BCoV and DcCoV UAE-HKU23 strains/BCoV/HCoV-OC43; and a plot of effective number of codons against use of G or C at third position of codons of ORF1ab, HE, S, E, M and N genes in betacoronavirus lineage A. [file 13-1769-Techapp-s1.pdf]

Article DOI: http://dx.doi.org/10.3201/eid2004.131769

Novel Betacoronavirus in Dromedaries of the Middle East, 2013

Technical Appendix

Technical Appendix Table 1. Coding potential and putative TRSs of the genomes of DcCoV UAE-HKU23

CoV, strain no., ORF	Location, nt	Length, nt	Length, aa	Frame	Putative TRS, TRS location, nt	Putative TRS, TRS sequence(s), distance in bases to AUG*
DcCoV UAE-HKU23 (265F)						
1ab	215–21498	21284	7094	+2,+1	68	**UCUAAAC**(140)AUG
NS2	21508–22344	837	278	+1	21494	**UCUAAAC**UUUAAGAAUG
HE	22356–23630	1275	424	+3	22340	**ACUAAAC**UCAGUGAAAAUG
S	23645–27745	4101	1366	+2	23638	**UCUAAAC**AUG
NS5	28114–28443	330	109	+1	28056	**GGUGAAC**(51)AUG
E	28430–28684	255	84	+2	28300	**UCCAAAC**(123)AUG
M	28699–29391	693	230	+1	28689	**UCCAAAC**AUUAUG
N	29401–30747	1347	448	+1	29387	**UCUAAAC**UUUAAGGAUG
N2	29462–30085	624	207	+2		
DcCoV UAE-HKU23 (362F)						
1ab	215–21498	21284	7094	+2,+1	68	**UCUAAAC**(140)AUG
NS2	21508–22344	837	278	+1	21494	**UCUAAAC**UUUAAGAAUG
HE	22356–23630	1275	424	+3	22340	**ACUAAAC**UCAGUGAAAAUG
S	23645–27745	4101	1366	+2	23638	**UCUAAAC**AUG
NS5	28114–28443	330	109	+1	28056	**GGUGAAC**(51)AUG
E	28430–28684	255	84	+2	28300	**UCCAAAC**(123)AUG
M	28699–29391	693	230	+1	28689	**UCCAAAC**AUUAUG
N	29401–30747	1347	448	+1	29387	**UCUAAAC**UUUAAGGAUG
N2	29462–30085	624	207	+2		
DcCoV UAE-HKU23 (368F)						
1ab	215–21498	21284	7094	+2,+1	68	**UCUAAAC**(140)AUG
NS2	21508–22344	837	278	+1	21494	**UCUAAAC**UUUAAGAAUG
HE	22356–23630	1275	424	+3	22340	**ACUAAAC**UCAGUGAAAAUG
S	23645–27745	4101	1366	+2	23638	**UCUAAAC**AUG
NS5	28114–28443	330	109	+1	28056	**GGUGAAC**(51)AUG
E	28430–28684	255	84	+2	28300	**UCCAAAC**(123)AUG
M	28699–29391	693	230	+1	28689	**UCCAAAC**AUUAUG
N	29401–30747	1347	448	+1	29387	**UCUAAAC**UUUAAGGAUG
N2	29462–30085	624	207	+2		

*Boldface indicates putative TRS sequences. TRS, transcription regulatory sequences; DcCoV, dromedary camel coronavirus; CoV, cornonavirus.

Technical Appendix Table 2. Characteristics of putative nonstructural proteins of ORF1ab in DcCoV UAE-HKU23*

nsp	Putative function/domain	First amino acid residue^{position}	Last amino acid residue^{position}	Length, aa	Length of corresponding protein in HCoV-OC43, BCoV, ECoV, and PHEV
nsp1	Leader protein	M^{1}	G^{246}	246	246 (244 in ECoV)
nsp2	MHV p65-like protein	V^{247}	A^{851}	605	605 (601 in ECoV)
nsp3	PL1pro, PL2pro, AC, ADRP, HD	G^{852}	G^{2750}	1899	1899 (1951 in ECoV)
nsp4	HD	A^{2751}	Q^{3246}	496	496
nsp5	3CLpro	S^{3247}	Q^{3549}	303	303
nsp6	HD	S^{3550}	Q^{3836}	287	287
nsp7	Unknown	S^{3837}	Q^{3925}	89	89
nsp8	Unknown	A^{3926}	Q^{4122}	197	197
nsp9	Unknown	N^{4123}	Q^{4232}	110	110
nsp10	Unknown	A^{4233}	Q^{4369}	137	137
nsp11	Unknown (short peptide at the end of ORF1a)	S^{4370}	V^{4383}	14	14
nsp12	RdRp	S^{4370}	Q^{5297}	928	928
nsp13	Hel	S^{5298}	Q^{5900}	603	603 (599 in ECoV)
nsp14	ExoN	C^{5901}	Q^{6421}	521	521
nsp15	XendoU	S^{6422}	Q^{6795}	374	375 (366 in ECoV and 374 in BCoV)
nsp16	2'-O-MT	A^{6796}	I^{7094}	299	299

*ORF, open-reading frame; DcCoV, dromedary camel coronavirus; PL1pro, PL2pro, papain-like protease 1 and papain-like protease 2; AC, acidic domain; ADRP, adenosine diphosphate-ribose 1"-phosphatase; HD, hydrophobic domain; 3CLpro, 3C-like protease; RdRp, RNA-dependent RNA polymerase; Hel, helicase; ExoN, 3′-to-5′ exonuclease; XendoU, poly(U)-specific endoribonuclease; 2'-O-MT, 2'-*O*-ribose methyltransferase.

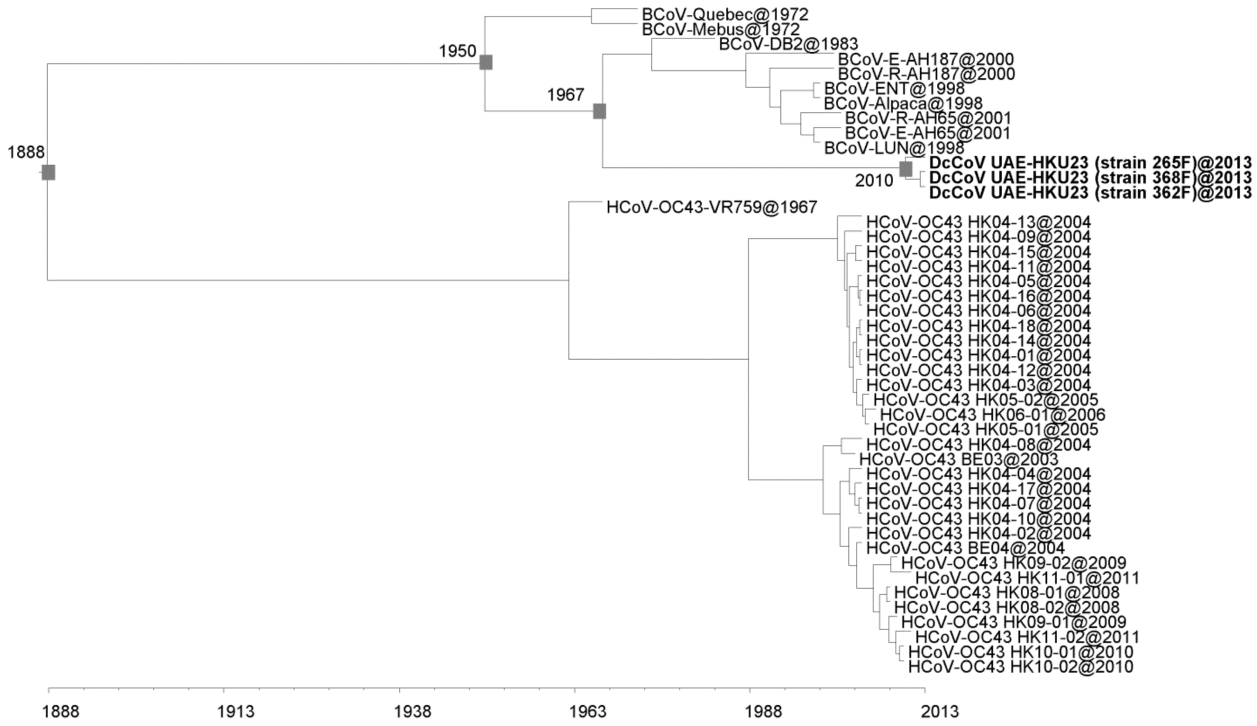

Technical Appendix Figure 1. Estimation of tMRCA (time to most common ancestor) of dromedary camel coronavirus (DcCoV) UAE-HKU23 strains, DcCoV UAE-HKU23 strains/bat CoV, and DcCoV UAE-HKU23 strains/bat CoV/HCoV-OC43 based on the RNA-dependent RNA polymerase gene. The mean estimated dates are labeled and are represented by gray squares. The taxa are labeled with their sampling years.

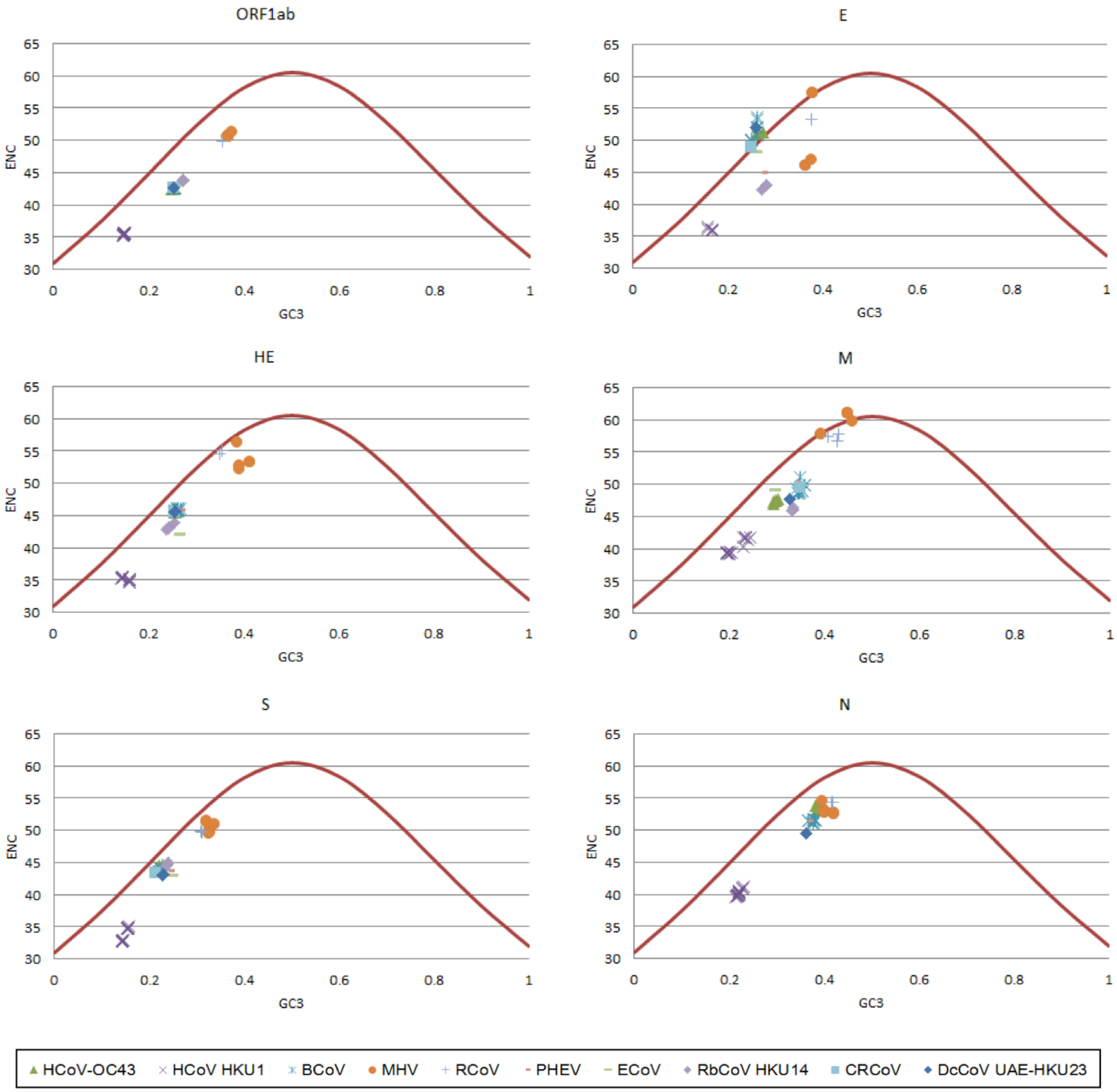

Technical Appendix Figure 2. A plot of effective number of codons (ENC) against use of G or C at third position of codons (GC3) of open-reading frame (ORF)1ab, HE, S, E, M, and N genes in lineage A of *Betacoronavirus*. The continuous curve plots the relationship between ENC and GC3 in the absence of selection.
